# The S68G polymorphism is a compensatory mutation associated with the drug resistance mutation K65R in CRF01_AE strains

**DOI:** 10.1186/s12879-020-4836-z

**Published:** 2020-02-11

**Authors:** Shengjia Li, Jinming Ouyang, Bin Zhao, Minghui An, Lin Wang, Haibo Ding, Min Zhang, Xiaoxu Han

**Affiliations:** 1 NHC Key Laboratory of AIDS Immunology (China Medical University), Department of Laboratory Medicine, The First Affiliated Hospital of China Medical University, Shenyang, 110001 China; 2 National Clinical Research Center for Laboratory Medicine, Shenyang, 110001 China; 3 Key Laboratory of AIDS Immunology, Chinese Academy of Medical Sciences, Shenyang, 110001 China; 4 Department of Laboratory Medicine, The First Affiliated Hospital of China Medical University, Shenyang, 110001 China

**Keywords:** CRF01_AE, Polymorphism, Drug resistance, Replication fitness

## Abstract

**Background:**

The rate of S68G mutation in human immunodeficiency virus type 1 (HIV-1) reverse transcriptase has increased and is closely related to the K65R mutation among CRF01_AE-infected patients who failed treatment. We aimed to explore the temporal association of S68G and K65R mutations and disclose the role of the former on susceptibility to nucleotide/nucleoside reverse transcriptase inhibitor (NRTI) and viral replication with the K65R double mutations among CRF01_AE-infected patients who failed treatment.

**Methods:**

The occurrence of S68G and K65R mutations was evaluated among HIV-1 of various subtypes in the global HIV Drug Resistance Database. The temporal association of S68G and K65R mutations was analyzed through next-generation sequencing in four CRF01_AE-infected patients who failed treatment with tenofovir/lamivudine/efavirenz. The impact of the S68G mutation on susceptibility to NRTI and replication fitness was analyzed using pseudovirus phenotypic resistance assays and growth competition assays, respectively.

**Results:**

The frequency of the S68G mutation increased by 1.4–9.7% in almost all HIV subtypes and circulating recombinant forms in treatment-experienced patients, except subtype F. The S68G mutation often occurred in conjunction with the K65R mutation among RTI-treated patients, with frequencies ranging 21.1–61.7% in various subtypes. Next-generation sequencing revealed that the S68G mutation occurred following the K65R mutation in three of the four CRF01_AE-infected patients. In these three patients, there was no significant change detected in the half maximal inhibitory concentration for zidovudine, tenofovir, or lamivudine between the K65R and K65R/S68G mutations, as demonstrated by the phenotypic resistance assays. Virus stocks of the K65R and K65R/S68G mutations were mixed with 4:6, 1:1, and 9:1 and cultured for 13 days, the K65R/S68G mutants outgrew those of the K65R mutants irrespective of the input ratio.

**Conclusions:**

S68G may be a natural polymorphism and compensatory mutation of K65R selected by NRTIs in the CRF01_AE strain of HIV-1. This mutation does not affect susceptibility to NRTI; however, it improves the replication fitness of K65R mutants. This study deciphers the role of the S68G mutation in the HIV reverse transcriptase of the CRF01_AE strain and provides new evidence for the interpretation of drug-resistant mutations in non-B subtypes of HIV-1.

## Background

Interpretation of human immunodeficiency virus type 1 (HIV-1) drug resistance mutations (DRMs) is mainly based on phenotypic and genotypic studies of subtype-B, which accounts for ~ 12.1% of HIV infection cases worldwide [1]. Numerous patients infected with non-B subtype HIV-1 in developing countries receiving antiretroviral therapy (ART) require more precise interpretation of DRMs [2, 3]. The high genetic diversity of HIV-1 poses great challenges to the interpretation of the genotyping of drug resistance.

Several studies have suggested that DRMs may not be fully consistent among different subtypes of HIV-1. For instance, HIV-1 strains of different subtypes can develop various “signature” DRMs under the same ART regimen [4, 5]. Some studies have suggested that natural polymorphisms of the non-B subtypes of HIV-1 change their susceptibility to ART independently or in combination with other mutations [6, 7]. Additional studies are warranted to elucidate the role of natural polymorphisms and certain treatment-associated mutations in the non-B subtypes of HIV-1.

CRF01_AE is the first circulating recombinant form of HIV-1 identified worldwide. It originated from Central Africa, and accounts for 4.6% of the total number of HIV-1 infections worldwide [8, 9]. CRF01_AE may have distinctive characteristics on pathways related to natural polymorphisms and the development of DRM [10–12]. A preliminary study on CRF01_AE-infected patients, conducted in China by our research team, found that the frequency of the S68G mutation showed a 5.5% increase in patients who failed treatment. Moreover, the S68G mutation demonstrated a close link with the K65R mutation (unpublished data). The role of the S68G mutation in drug resistance and its relationship with the K65R mutation has yet to be elucidated. Thus, we conducted this study to: 1) explore the frequency of S68G mutation and K65R/S68G double mutation occurrence among various subtypes of HIV-1 worldwide; 2) ascertain the temporal association of S68G and K65R mutations among patients infected with CRF01_AE who failed treatment; and 3) understand the role of the S68G mutation in susceptibility to NRTI and viral replication when combined with the K65R mutation.

## Methods

### Occurrence of S68G and K65R/S68G mutations among various subtypes of HIV-1

The prevalence of S68G mutation and K65R/S68G double mutation among various subtypes of HIV-1 was analyzed in reverse transcriptase inhibitor (RTI)-naive and RTI-treated individuals from the HIV Drug Resistance Database of Stanford University (https://hivdb.stanford.edu/cgi-bin/MutPrevBySubtypeRx.cgi; version of 19 April 2019). For patients having more than one isolates with the same mutation are counted once. Mutations occurring in ≥1% and at least two individuals were included in the analyses. All sequences with a mixture of mutations were excluded from the analyses.

### Study participants and sample collection

Four patients infected with CRF01_AE who failed treatment and carried the K65R and S68G double mutations were selected from a large-scale long-term ART cohort at the First Affiliated Hospital, China Medical University (Shenyang, China) (Additional file 1: Figure S1). The patient identification numbers were 301635, 301770, 301844, and 302335. The treatment regimens were tenofovir (TDF), lamivudine (3TC), and efavirenz. Genotypic testing for drug resistance and the viral load of plasma was performed at baseline, and at 6, 7, 10, and 12 months after treatment. Thereafter, all four patients changed treatment regimens. Among the four patients, 301635 and 302335 were followed up four times within 6–7 months post ART, while 301770 and 301844 were followed up five times within 10–12 months post ART. The study protocol was approved by the Ethics Committee of the First Affiliated Hospital of China Medical University. Written informed consent was provided by all patients.

### Extraction, amplification, and sequencing of HIV-1 DNA

Viral RNA was extracted from plasma using a QIAamp® RNA Blood Mini kit (Qiagen, Stanford, VA, USA). The partial pol gene (145 bp) was amplified using primers 503b-F (5′-CAAAAATTGGGCCTGAAAATCCATA-3′) and 52r-R (5′-TGTGGTATTCCTAATTGAACTTCCCA-3′) through nested polymerase chain reaction (PCR). PCR products were purified with an Agencourt AMPure™ kit (Beckman Coulter, Fullerton, CA, USA) and quantified using a Qubit® dsDNA BR Assay kit (Life Technologies, Carlsbad, CA, USA). Subsequently, the PCR products were evaluated with a Bioanalyzer (2100; Agilent Technologies, Santa Clara, CA, USA) to control the size of the amplified fragments, and a library was constructed based on an input of 130–150 ng. A TruSeq™ Nano DNA HT Sample Preparation kit was employed for the construction of a DNA library (Illumina, San Diego, CA, USA). The quality of library construction was monitored using a DNA 7500 kit (Agilent Technologies). The standardized libraries were incorporated into 50% quality-controlled PHIX Libraries (Miseq phix control v3; Illumina). Sequencing was performed after the qualification of all operations.

The FASTQC (version 0.11.5) software was used to evaluate the sequencing quality of the paired-end FASTQ sequence files. MiSeq data analysis was performed using HyDRA Web, an open web portal that offers an automated pipeline for the analysis of next-generation sequencing-derived data related to HIV drug resistance. Advanced options for data quality assurance, filtering, variant calling, and reporting thresholds were modified to customize the analysis. Reads were filtered using a minimum quality score of Q30 and 50 bp in length, an error rate of 0.0021 for the MiSeq platform, a minimum variant quality of Q30, a minimum read depth of 100×, and a minimum allele count of five. HIV-DRMs detected with a frequency > 1% were reported based on the default HyDRA Web Mutation Database. This database is a combination of the Stanford 2015 list of HIV-1 DRMs with added annotations from the World Health Organization 2009 list of mutations for the surveillance of transmitted HIV drug resistance [13].

### Construction of a mutant clone and phenotypic resistance assay

Protease (PR) genes (codons 1–99) and reverse transcriptase (RT) genes (codons 1–240) were amplified from viral RNA obtained from patients infected with CRF01_AE using nested PCR using primers Round2-F (5′-ATAGCCAAAAATTGCAGGGCCCCTAGRAAAAAG-3′) and Round2-R (5′-GTCCTTCCTTTCCACATTTCCA-3′). Patient-derived PCR products containing the K65R/S68G double mutations were cloned into a pNL4–3-ΔE-Luc plasmid through a ApaI/AgeI double-enzyme digestion and T4 ligation strategy to produce the pNL4–3-ΔE-Luc K65R/S68G clone. Subsequently, the S68G mutation was reversed to wild-type through in vitro site-directed mutagenesis using primers G68S-F (5′-AAGAGAAAGGACAGTACCAAATGGAGAAAG-3′) and G68S-R(5′-TCTCCATTTGGTACTGTCCTTTCTCTTTAT-3′) to produce the pNL4–3-ΔE-Luc K65R clone. Pseudoviruses were packaged in 293 T cells with plasmid VSVG and co-transfection of the K65R/S68G clone or K65R/S68G clone [14]. The titers of viral stocks were determined according to the Spearman–Karber method [15].

The susceptibility of the virus to AZT, 3TC, and TDF was calculated as the half maximal inhibitory concentration (IC50). All experiments were conducted in triplicate. The IC50 of the mutant virus was compared with that of the fully susceptible virus (wild-type pseudovirus NL4–3-ΔE-Luc). The degree of resistance of the mutant pseudoviruses was determined by calculating the fold change (FC) in IC50 compared with that of the wild-type pseudoviruses.

### Growth competition assay

PR genes and RT genes of the pNL4–3-wildtype plasmid were replaced by patient-derived genes containing K65R/S68G double mutations through an ApaI/AgeI double-enzyme digestion and T4 ligation strategy to produce the pNL4–3-wildtype K65R/S68G mutant infectious clone. Subsequently, the S68G mutation was reverted to wild type through in vitro site-directed mutagenesis to produce the pNL4–3-wildtype K65R mutant infectious clone. The K65R mutant and K65R/S68G mutant viral stocks were produced via transfection of 293 T cells, and cultured at 37 °C in an atmosphere of 5% CO_2_ for 48 h. The supernatant was collected for viral titration. The K65R/S68G mutants and K65R mutants viral stocks were diluted and mixed at a 4:6, 1:1, and 9:1 ratio, respectively. Peripheral-blood mononuclear cells (3 × 10^5^) obtained from healthy donors were infected with the same virus titers (multiplicity of infection = 0.05) from each of the competing viruses. All experiments were conducted in triplicate. Viral fitness was determined through full pairwise competitions between all combinations of viruses. On days 3, 5, 7, 10, and 13, half of the culture supernatant was harvested, and viral RNA was extracted. The viral pol gene was amplified and sequenced for Sanger sequencing. The ChromatQuan Internet tool was used to calculate the viral ratio at each time point [16].

### Statistical analysis

The chi-squared test was used to evaluate the frequency of S68G mutation between RTI-naive and -treated individuals, and the K65R/S68G double mutation among HIV-1 subtypes. The nonparametric t-test was used to analyze differences in FC between K65R alone and K65R/S68G double mutants. A *p* < 0.05 denoted statistical significance.

## Results

### Frequency of the S68G mutation increased significantly in RTI-treated patients and commonly co-occurred with the K65R mutation among CRF01_AE strains

A total of 172,639 RTI-naive and RTI-treated individuals infected with HIV-1 in the HIV Drug Resistance Database of Stanford University were analyzed in this study: subtype A (*n* = 12,415), subtype B (*n* = 83,006), subtype C (*n* = 36,436), subtype D (*n* = 3705), subtype F (*n* = 2418), subtype G (*n* = 4638), CRF01_AE (*n* = 20,507), and CRF02_AG (*n* = 9514). Prior to ART, the lowest and highest prevalence of the S68G mutation were reported in subtype G (1.3%, *n* = 1564) and CRF01_AE (7.3%, *n* = 14,336), respectively. Among RTI-treated patients, the prevalence of the S68G mutation increased significantly in almost all subtypes (*p* < 0.05), except subtype F that exhibited a slight decrease. CRF01_AE viruses demonstrated a 9.7% increase in S68G mutation between RTI-treated and RTI-naive individuals, the greatest increase observed among all subtypes (Additional file 2: Table S1). Surprisingly, the S68G mutation showed a high tendency to coincide with the K65R mutation in various subtypes. The prevalence of the K65R/S68G double mutation among RTI-treated patients of various subtypes ranged from 21.1% (4/19) in subtype F to 61.7% (142/230) in CRF01_AE (Additional file 3: Table S2).

### S68G mutation is often selected on the basis of the K65R mutation

**Fig. 1:**
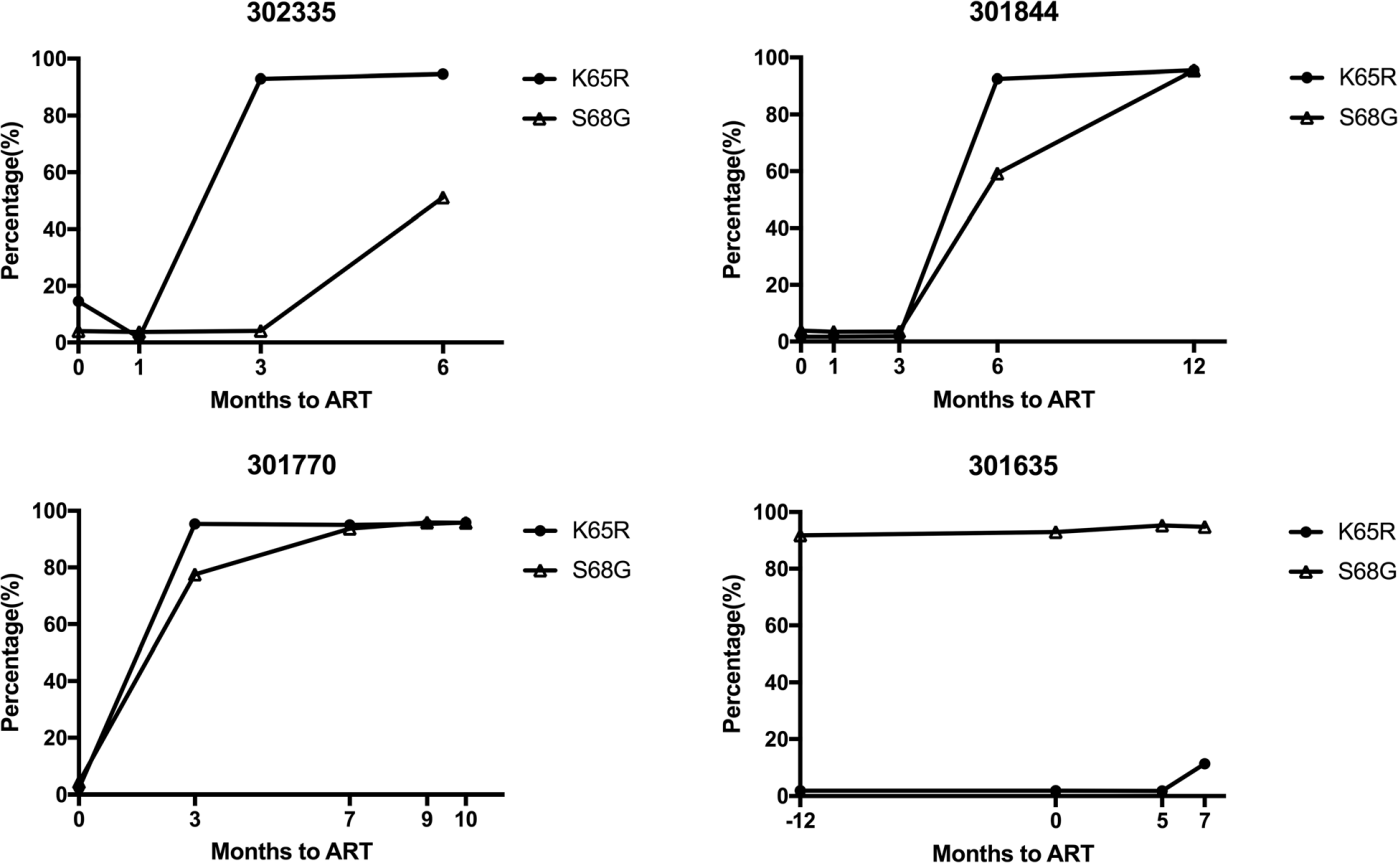
Temporal association of S68G and K65R mutations in individuals infected with CRF01_AE during antiretroviral treatment. 302335, 301844, 301770, and 301635 were four patients infected with CRF01_AE, in whom both the S68G and K65R mutations were detected after treatment failure. The pol sequences were studied in longitudinal plasma samples using next-generation sequencing. The black circle and triangle represent the percentages of K65R and S68G quasispecies, respectively

An average of 260,000 high quality reads (> Q30) were obtained from each of the four cases, which comprehensively reflected the sequence characteristics of the mutant. Among the three patients infected with CRF01_AE not carrying the natural polymorphism S68G prior to ART, The K65R mutation was detected in patient-302335 as the dominant mutant quasispecies 3 months post-ART, accounting for 92.99%, which remained at a high level (94.63%) when tested 6-months post-ART; at that time, the S68G mutation was detected among 51.14% quasispecies. The other two patients (301770 and 301844) had the predominant quasispecies carrying the K65R mutation at 3–6 months post ART (accounting for 95.38 and 92.51%, respectively). The S68G mutation occurred at 77.52 and 59.19% respectively. In further follow-up time points (at 7–12 months post ART), almost all quasispecies carried the K65R/S68G double mutation in these two patients. Unlike the above three patients, patient 301635 carried the S68G mutation as the predominant natural polymorphism, appearing in > 90% of the quasispecies prior to ART. However, the K65R mutation was not detected until 7 months post ART, when only 11.28% of the quasispecies were found to have the K65R mutation (Fig. 1).

### S68G mutation does not decrease the drug susceptibility of the K65R mutation to AZT, TDF, or 3TC

**Fig. 2:**
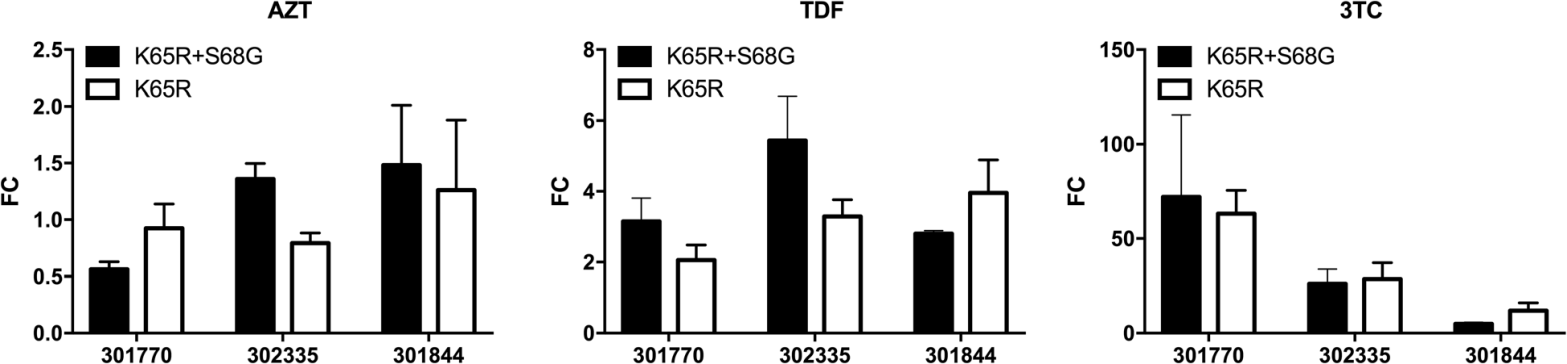
Pseudovirus-based phenotypic resistance assay for AZT, TDF, and 3TC. Black columns and white columns represent the K65R/S68G double mutation and K65R mutation pseudoviruses with mutant pol gene sequences obtained from patients, respectively. FC, fold change in IC50 compared with the wild-type pseudoviruses; AZT, zidovudine; TDF, tenofovir; 3TC, lamivudine. Data were averaged from at least three independent experiments

The frequency of the K65R mutation in patient 301635 was insufficient to select clones of the K65R/S68G double mutation; hence, this patient was not included in subsequent analyses. Therefore, clones of the K65R/S68G double mutation and K65R mutation from the other three patients were used for analyses of phenotypic drug resistance. The viruses harboring the K65R/S68G double mutation in patients 301770, 301844, and 302335 exhibited a FC to AZT of 0.562 ± 0.067, 1.483 ± 0.529, and 1.359 ± 0.137, respectively. These values were not significantly different from those of the K65R mutation (FC: 0.925 ± 0.214, 1.262 ± 0.618, and 0.793 ± 0.09, respectively; *p* > 0.05). Moreover, there were no significant changes observed in the degree of resistance to 3TC or TDF (Fig. 2).

### S68G mutation compensated for the replication fitness of the K65R mutation

**Fig. 3:**
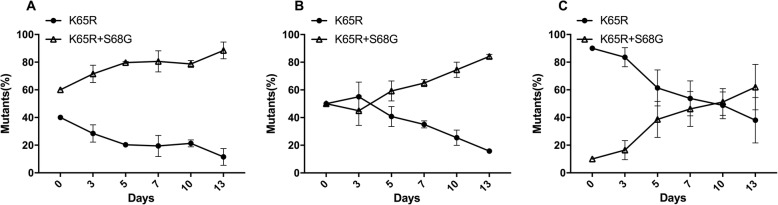
Growth competition assay for the K65R and K65R/S68G infectious clones. The infectious clones of K65R and K65R/S68G obtained from patients were co-cultured for 13 days in PBMCs with the following input ratios for K65R vs. K65R/S68G: 4:6 (**a**), 1:1 (**b**), and 9:1 (**c**). Viral RNA from culture supernatants was used to amplify target regions of pol genes, and these regions were subsequently sequenced. ChromatQuan (http://indra.mullins.microbiol.washington.edu/cgi-bin/chromatquant.cgi) was used to calculate the viral percentages at each time point. The average of three independent experiments is shown, with error bars representing the SD

Virus stocks of the K65R and K65R/S68G mutations were mixed with different input ratios and cultured for 13 days. At the end of culture, the K65R/S68G double mutation strains outgrew those of the K65R mutation irrespective of the input ratio. In the 4:6 input group, the composition of K65R/S68G mutants increased from 60 to 88%. In the 1:1 input group, the composition of K65R/S68G mutants increased from 50 to 84%. In the 9:1 input group, the composition of K65R/S68G mutants increased from 10 to 62% (Fig. 3).

## Discussion

In this study, we found that the S68G mutation was a common natural polymorphism in various subtypes of HIV-1, predominantly among CRF01_AE strains. The S68G mutation often occurred following the K65R mutation in patients infected with CRF01_AE who failed treatment. However, the pre-existence of the S68G natural polymorphism may not accelerate the occurrence of the K65R mutation. Although S68G did not directly alter the drug susceptibility of the virus, it compensated for the loss of replication fitness associated with the K65R mutation in CRF01_AE HIV-1.

We found that the mutation rate of S68G increased in almost all HIV-1 subtypes in drug-experienced populations, with the greatest increase noted among CRF01_AE strains. This finding is supported by several studies on non-subtype B HIV-1 [17, 18]. Based on the available evidence, it is suggested that S68G may be new mutation associated with the development of drug resistance.

Moreover, previous studies showed that patients infected with subtype B and non-B subtype HIV-1, who failed ART and developed the K65R mutation, also acquired the S68G mutation [18–22]. However, the unexpected high frequency of the S68G mutation in combination with the K65R mutation in the CRF01_AE strain has not been previously reported. Several studies have examined the evolution of the K65R and S68G mutations using standard testing for HIV drug resistance or molecular clones. However, the conclusions drawn from these investigations have been controversial. Ross et al. reported that mutations at position 68 followed the initial selection for K65R and M184 V mutations [23]. Roge et al. found that five patients with the wild-type virus at baseline developed K65R at the time of failure, and four of those patients also acquired the S68G mutation [20]. However, Wirden et al. found that the S68G mutant was the dominant quasispecies, whereas K65R/S68G double mutant was the minor quasispecies. This finding suggested that the S68G mutation may not be linked to the K65R mutation [24]. In the aforementioned studies, variants with a frequency < 20% of the viral population could hardly be detected due to the limitation of Sanger sequencing, molecular clones, and PCR bias [25]. In this study, we performed next-generation sequencing to explore the evolutionary relationship between K65R and S68G mutations using longitudinal samples. This approach can detect viral quasispecies with frequencies as low as 1%, and generate more data than Sanger sequencing (1000s–10000s magnitude) [26]. Therefore, we disclosed the temporal association between these two mutations among patients infected with CRF01_AE who failed treatment. Our results demonstrated that the frequency of the K65R/S68G double mutation was lower than that of the K65R mutation in vivo. Moreover, a time lag was detected between the occurrence of the S68G and K65R mutations. Hence, the K65R mutation may be induced first as the major mutation under the pressure of ART, while S68G may be an accessory mutation of K65R that is subsequently caused on the basis of the K65R mutation. We sought to investigate whether the preexistence of the S68G natural polymorphism prior to treatment could assist the development of the K65R mutation. We noticed that, in one patient with > 90% quasispecies carrying the S68G mutation prior to treatment, the K65R mutation was not detected until 7 months post ART. This timing was later than that observed for the remaining three patients not carrying the S68G polymorphism prior to treatment, suggesting that the S68G mutation may not accelerate the development of the K65R mutation.

We subsequently examined the phenotype of S68G. Svarovskaia evaluated the role of the S68G in combination with K65R in subtype B. The analysis revealed that S68G mutations partially restored the replication defect associated with the K65R mutation; however, it did not cause significant change in resistance of HIV-1 to NRTIs [27]. In this study, we focused on the phenotype of S68G in the CRF01_AE virus. CRF01_AE virus had an obvious genetic difference on the pol gene compared with that of the subtype B virus. In addition, it occurred markedly more often in both treatment-naive patients and those who failed treatment than that of the subtype B virus. We used patient-derived pol gene fragment replacement for the construction of the mutant plasmid instead of site-directed mutagenesis on the basis of the subtype B standard plasmid to maintain the genetic background of the pol gene of CRF01_AE. We disclosed the minor role of the K65R mutation with and without S68G in susceptibility to NRTI. However, the S68G mutation helps to compensate the loss of replicative fitness, which is similar to that of subtype B.

The K65 residue is located on the key position of the deoxynucleoside triphosphate binding pocket and associated with the development of resistance to nucleoside analogs and polymerase fidelity [28]. This residue confers resistance to numerous NRTIs by reducing the efficiency of NRTI incorporation. It has also been speculated that the K65R substitution improves the fidelity of the polymerase, thereby reducing the occurrence of several types of RT errors, including substitution and frameshift mutations [29–31]. It has been reported that the increase in polymerase fidelity may render the viral population less able to adapt to drug treatment and immune surveillance, and reduce replication fitness [32]. Given the importance of K65 residues in the RT function of HIV-1, and the observed reduced fitness of the K65R mutant, the virus must evolve compensatory mutations against the impairment. With regard to patients who developed the K65R/S68G double mutation, restoration of the replication fitness of the virus through a compensatory mutation can help the virus survive under drug pressure, potentially leading to disease progression [33, 34]. Moreover, this adaptive evolution may shape HIV-1 at the population level, when these viruses contribute to epidemics through transmission. The resultant, potentially more virulent virus, could eventually modulate disease progression in newly infected hosts [35].

A limitation of this study is its small sample size. Owing to the high success rate of antiviral therapy in our cohort, only four patients with K65R/S68G double mutations were analyzed. Studies investigating a greater number of cases are warranted to elucidate the functional role of the S68G mutation. Nevertheless, this study provides evidence to aid the interpretation of DRMs in CRF01_AE strains.

## Conclusions

We deciphered the role of the S68G mutation in the HIV RT of CRF01_AE strains. Our study provides new evidence for the interpretation of DRMs in the CRF01_AE strain and non-B subtypes of HIV-1. The present study also suggests the presence of additional potential DRMs in the non-B subtypes of HIV-1. Further studies are required to evaluate the involvement of the HIV phenotypes in the development of drug resistance.

## Supplementary information

**Additional file 1: Figure S1.** Flowchart of patients included in this study.

**Additional file 2: Table S1.** Prevalence of the S68G mutation among HIV-1 strains of various subtypes in the Stanford University HIV Drug Resistance Database.

**Additional file 3: Table S2.** Frequency of mutation on site 68 of reverse transcriptase between different subtypes in K65R mutants.

## Data Availability

The datasets used and/or analyzed during the current study are available from the corresponding author upon reasonable request.

## Abbreviations

3TC: Lamivudine
ART: Antiretroviral therapy
AZT: Zidovudine
DRMs: Drug resistance mutations
HIV: Human immunodeficiency virus
NRTI: Nucleotide/nucleoside reverse transcriptase inhibitor
PCR: Polymerase chain reaction
TDF: Tenofovir
