# Supplementary figures and images for: The S68G polymorphism is a compensatory mutation associated with the drug resistance mutation K65R in CRF01_AE strains
Source: BMC Infect Dis. 2020 Feb 11;20:123. doi: 10.1186/s12879-020-4836-z (PMC7014709; doi:10.1186/s12879-020-4836-z)

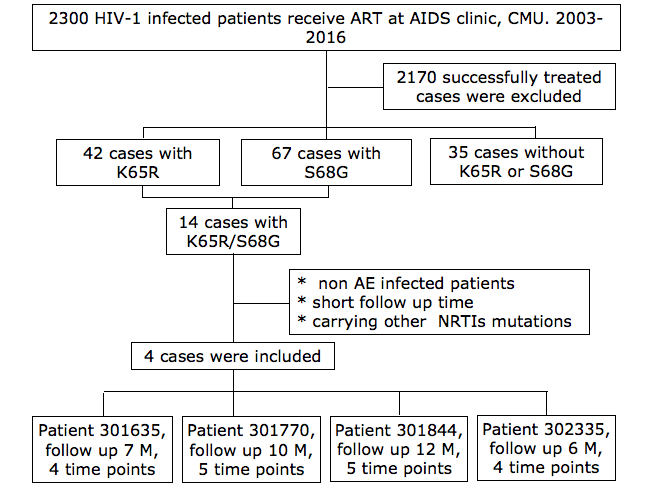

Supplement: Supplementary file 1 — Additional file 1: Figure S1. Flowchart of patients included in this study. [file 12879_2020_4836_MOESM1_ESM.tif]
