# Supplementary material for: The S68G polymorphism is a compensatory mutation associated with the drug resistance mutation K65R in CRF01_AE strains
Source: BMC Infect Dis. 2020 Feb 11;20:123. doi: 10.1186/s12879-020-4836-z (PMC7014709; doi:10.1186/s12879-020-4836-z)
Supplement: Supplementary file 2 — Additional file 2: Table S1. Prevalence of the S68G mutation among HIV-1 strains of various subtypes in the Stanford University HIV Drug Resistance Database. [file 12879_2020_4836_MOESM2_ESM.docx]

**Table S1.** Prevalence of the S68G mutation among HIV-1 strains of various subtypes in the Stanford University HIV Drug Resistance Database

| Subtype | RTI-naive  % / (n) | RTI-treated  % / (n) | Increase  (%) | P-value |
| --- | --- | --- | --- | --- |
| A | 1.9 (7089) | 6.1 (5326) | 4.2 | < 0.05 |
| B | 4.5 (46589) | 5.9 (36417) | 1.4 | < 0.05 |
| C | − (15809) | 4.8 (20627) | 4.8 | - |
| D | 1.6 (2023) | 5.9 (1682) | 4.3 | < 0.05 |
| F | 3.5 (1532) | 2.3 (886) | −1.2 | > 0.05 |
| G | 1.3 (1564) | 4.2 (3074) | 2.9 | < 0.05 |
| CRF01_AE | 7.3 (14336) | 17 (6171) | 9.7 | < 0.05 |
| CRF02_AG | 4.7 (5175) | 10 (4339) | 5.3 | < 0.05 |

RTI-naive patients had never been exposed to antiretroviral drugs, whereas RTI-treated patients were receiving RTI.

%, percentage of S68G mutation among various HIV-1 subtypes

n, number of isolates according to subtype and drug class exposure

HIV, human immunodeficiency virus

RTI, reverse transcriptase inhibitor
