# Supplementary material for: The S68G polymorphism is a compensatory mutation associated with the drug resistance mutation K65R in CRF01_AE strains
Source: BMC Infect Dis. 2020 Feb 11;20:123. doi: 10.1186/s12879-020-4836-z (PMC7014709; doi:10.1186/s12879-020-4836-z)
Supplement: Supplementary file 3 — Additional file 3: Table S2. Frequency of mutation on site 68 of reverse transcriptase between different subtypes in K65R mutants. [file 12879_2020_4836_MOESM3_ESM.docx]

Table S2. Frequency of mutation on site 68 of reverse transcriptase between different subtypes in K65R mutants

| Mutation | Subtype (%) | | | | | | | |
| --- | --- | --- | --- | --- | --- | --- | --- | --- |
|  | A (n = 214) | B (n = 748) | C (n = 1345) | D (n = 55) | F (n = 19) | G (n = 147) | CRF01_AE (n = 230) | CRF02_AG (n = 171) |
| G | 41.6 | 38.9 | 27.9 | 27.3 | 21.1 | 29.3 | 61.7 | 43.3 |
| N | 3.7 | 7 | 19.4 | 18.2 | 15.8 | 6.8 | 6.1 | 3.5 |
| D | 1.4 | 3.2 | 2.8 | 1.8 | – | – | 2.2 | 1.2 |
| K | 1.9 | 2.8 | 2.4 | 3.6 | – | 1.4 | 1.3 | 1.8 |
| R | 0.5 | 2.7 | 0.8 | 1.8 | – | 2.0 | 0.9 | 0.6 |
| Total | 49.1 | 54.6 | 53.3 | 52.7 | 36.9 | 39.5 | 72.2 | 50.4 |

n, number of isolates according to subtype and drug class exposure; G, glycine; N, asparagine; D, aspartic acid; K, lysine; R, arginine.

* All data derived from the Stanford University HIV Drug Resistance Database.
